# Web-Based Delivery of the Caregiving Essentials Course for Informal Caregivers of Older Adults in Ontario: Mixed Methods Evaluation Study

**DOI:** 10.2196/25671

**Published:** 2021-06-15

**Authors:** Shelley Rottenberg, Allison Williams

**Affiliations:** 1 School of Earth, Environment & Society McMaster University Hamilton, ON Canada

**Keywords:** informal caregivers, family caregivers, older adults, elder care, web-based intervention, online intervention, online course, health education, eHealth, evaluation

## Abstract

**Background:**

Many informal caregivers of older adults have limited time because of the number of responsibilities that their caregiving role entails. This population often experiences high levels of burden due to the stressful nature of their work and are vulnerable to developing negative psychological health outcomes. Easily accessible and flexible knowledge interventions are needed to alleviate the burden and stress experienced by this group.

**Objective:**

This study aims to evaluate the acceptability of the web-based delivery of the *Caregiving Essentials* course for informal caregivers of older adults. Both the strengths and limitations of using a web-based platform to provide information and resources were explored to see whether the method of delivery enhanced or hindered the overall course experience for participants.

**Methods:**

A mixed methodology of web-based pre- (n=111) and postcourse surveys (n=39) and telephone interviews (n=26) was used to collect both qualitative and quantitative data from participants. Individual interviews were also conducted with key stakeholders (n=6), and a focus group was conducted with nursing students (n=5) who were involved in the project.

**Results:**

The web-based delivery of the course provided participants with greater accessibility to the course because it allowed them to work independently through the modules at their own pace wherever and whenever. The discussion boards were also identified as a major strength because of the opportunity for social interaction and the sense of community that many felt through sharing their experiences. Some barriers to participation included age-related factors, issues with navigating aspects of the course, and concerns about privacy and anonymity. Some key suggestions included more engaging methods of web-based communication and the reorganization of the module content to reduce the amount of text and streamline information.

**Conclusions:**

The web-based delivery of *Caregiving Essentials* appeared to enhance the overall course experience by increasing accessibility and allowing participants to interact with the learning materials and other caregivers. The findings from this evaluation can be used to create and improve the web-based delivery of both the current and emerging interventions for caregivers.

## Introduction

### Background

Informal caregivers are those who provide unpaid care to someone with at least 1 short- or long-term health condition or disability [1]. Family members often take on these roles and act as the primary support systems, especially when the care recipient is an older adult [2]. Caregiving responsibilities involve identifying and addressing needs through direct care provision, care management, or a combination of both [3]. Traditionally, this work was done by spouses, daughters, or daughters-in-law, given the gendered nature of caregiving work [3]. As of 2012, most informal caregivers in Canada were women (53%) [4], and in 2018, most (61%) were aged between 45 years and 64 years, and almost half (47%) were the adult children or children-in-law of their care recipients [5].

There is a growing number of Canadians engaging in unpaid, informal care work [6]. This is largely caused by Canada’s aging population, which is an increasing demographic trend. Another contributing factor is the shift in the responsibility of care from institutions to communities and families. In 2018, 7.8 million Canadians reported having provided care to a family member or friend with a long-term health condition, disability, or aging need [5]. The number of Canadians who will need to be cared for is expected to double over the next 30 years [7]. Caregivers identified age-related needs as the single most common problem for which they required help [6]. Therefore, the percentage of the population engaging in informal care work is likely to continue to grow in the coming years.

The informal caregiving of an older adult is often overwhelming and stressful because of the diversity of responsibilities and the unpredictable nature of the work. It usually calls for a mixture of emotional, physical, psychological, social, and financial support from the caregiver on a regular basis [8]. In addition, the role requires a knowledge base and skill set that many family members and friends are unequipped with at the onset of their caregiving journey [3,9]. In many cases, family caregivers must learn information and seek out resources along the way, which further adds to the burden they experience. Sometimes, people may be unexpectedly thrown into the role of caregiving when health complications arise suddenly in a friend or family member. In some cases, informal caregivers assume the role because it is seen as a family obligation [10]. Consequently, it is crucial that caregivers have access to proper support and resources to help alleviate stress and potential negative health outcomes.

However, the availability and accessibility of formal care services are not equally distributed across space [1]. Rural and remote locations have little to no services to support a family member providing care for an older adult. Even for the resources that do exist in rural areas, limitations such as distance and money may prevent caregivers from accessing them. When informal caregivers are isolated from the health care system and trained professionals, they experience more unmet tangible needs and, thus, more burden. This accessibility gap between urban and rural caregivers can result in differential health status among the care recipients [11]. Consequently, there is a significant need for more easily accessible information to be available for informal caregivers of older adults, irrespective of where they live.

### Previous Work

Many interventions have been implemented over the years to meet the needs of informal caregivers of older adults. The literature shows that interventions that are individually tailored and have multiple components are the most effective types for this population [12,13]. Research has indicated that interventions with multiple components have led to stronger physical and mental health benefits for participants when compared with single-component programs [10]. Psychoeducational interventions that can be personalized allow for more significant effects because of targeted intervention delivery [14].

Although traditional face-to-face interventions are more common, eHealth interventions are growing in popularity. The number of people seeking web-based support is increasing [15], as is the number of internet users who are older adults [16]. Therefore, web-based interventions fit with the contemporary behavior of many informal caregivers today. In addition, they allow for both individualization and the use of multiple components. The 4 major components of internet-based interventions are (1) content, (2) multimedia, (3) interactive web-based activities, and (4) guidance and supportive feedback [17].

Several web-based interventions have been conducted for different types of informal caregivers, demonstrating the feasibility of using this mode of delivery. In a systematic review, the results indicated that internet interventions can improve various aspects of caregiver well-being [16]. Similarly, in another systematic review, the impact of web-based interventions for caregivers was deemed to be clearly positive, with improvements in self-efficacy, anxiety, and depression observed [18]. Other promising web-based intervention outcomes have been seen, such as a reduction in caregiver burden [19], an increase in social support and role awareness [20], and a greater intention to access help from others [12].

In terms of the acceptability of web-based delivery, caregivers responded positively to initiatives involving web-based education and internet support groups [21]. For example, in a pilot study on a videoconferencing intervention, 95% of the family caregiver participants reported that using computers for group meetings was either very positive or moderately positive [22]. Moreover, it has been shown that internet-based interventions for informal caregivers are acceptable and just as effective as the conventional face-to-face interventions [18].

Due to service access limitations, informal caregivers may not want or be able to use formal care services and other resources. Therefore, internet interventions can provide education and support to informal caregivers facing participation barriers [22]. Furthermore, as web-based interventions are generally more cost-effective and accessible to informal caregivers than in-person interventions, they present promising opportunities for scalability [23].

Accessibility and asynchronism, which is the lack of simultaneous occurrence, were the 2 advantages identified by participants regarding the web-based modality of a training program [14]. Participants in that intervention also emphasized the importance of interacting with other caregivers because it reduced social isolation [14]. In another study on internet-based support, the findings revealed that anonymity, asynchronism, and connectivity were the main advantages of computer-mediated communication [15]. In terms of connecting with other caregivers, participants were more engaged and experienced more benefits when the intervention type was more interactive [10]. One systematic review observed that interactive web-based activities paired with the provision of human support were helpful in enhancing the psychological well-being of caregivers [17].

Despite the many positive outcomes of internet-based interventions and the several strengths of web-based delivery, there is a lack of randomized controlled trials [23]. The heterogeneity in intervention design, methodologies, outcomes, and participant characteristics, among others, makes cross comparison unattainable. More rigorous study designs and stronger methods would allow for more robust conclusions on the efficacy of such interventions for informal caregivers of older adults [21]. Further research should be conducted to determine which types of web-based interventions work best for which types of caregivers [24].

### Context and Goal of the Study

The *Caregiving Essentials* course [25] is a no-cost knowledge intervention hosted on Desire2Learn. The self-paced 8-week course was created by team members from the McMaster Centre for Continuing Education, the McMaster Institute for Research on Aging, and the Thrive Group to meet the needs of informal caregivers for practical, accessible, and timely information [26]. The web-based course was launched with 2 pilot offerings, one in the fall of 2018 and the other in the winter of 2019. The course aimed to enhance caregivers’ knowledge and confidence regarding health care issues pertaining to older adults, improve caregivers’ understanding and access to health and community care systems, and increase caregivers’ personal health and well-being.

*Caregiving Essentials* includes 4 stand-alone modules, each with a specific focus, and a resources module that features carefully selected materials. The module titles are as follows: (1) You and the Caregiver Role; (2) Your Caregiver Toolbox: Health and Medical Fundamentals; (3) Navigating Complex Systems and Getting the Support You Need; (4) The Importance of Looking After You; and (5) Resources. The curriculum offers users reliable, relevant, and up-to-date information on key topics related to the caregiving journey. Content was gathered from credible sources, such as the McMaster Optimal Aging Portal [27], and was reviewed by subject matter experts. Following each module, participants can assess their level of knowledge and understanding by completing self-check quizzes. The Caregiver Action Plan, a digital guide created to supplement the course, is linked to certain exercises woven across the modules. It provides participants with an individualized and practical resource at the end of the course. There are also prompts within each module that are connected to discussion board threads, where participants can engage with each other on the web.

The aim of this study is to evaluate the acceptability of the web-based delivery of the *Caregiving Essentials* course for informal caregivers of older adults. To determine whether the web-based delivery was well received and its impact on the usability of the course overall, those involved in the project were asked to provide feedback after course completion. Strengths, limitations, and areas of improvement related to the web-based functionality were identified by participants to determine whether the method of delivery enhanced or hindered different aspects of the user experience.

## Methods

### Recruitment

The inclusion criteria for the *Caregiving Essentials* course specified that participants must be the primary caregiver to an older adult (65 years or older) who is still living at home. Recruitment strategies targeted people residing in Hamilton, Sudbury, or Timmins for the fall course offering, and then efforts were expanded to anywhere in Ontario for the winter course offering. Participants were recruited using various community partner networks, such as long-term care homes, respite relief services, senior community centers, and academic institutions. The participants involved in the course evaluation were informal caregivers of older adults who had finished the majority of the module material by the official course end date. Participation in the evaluation was not a compulsory component of the course; therefore, data were only collected from those who were willing to offer their feedback (Table 1). The course users who completed all elements of the evaluation (pre- and postcourse surveys and a telephone interview) received a Can $20.00 (US $16.58) Tim Hortons gift card as a token of appreciation.

**Table 1:** Caregiver participants’ engagement numbers.

Pilot course offering	Recruitment inquiries	Course registrants	Precourse surveys completed	Postcourse surveys completed	Telephone interviews conducted
Total, n	315	140	111	39	26
Fall 2018, n (%)	150 (47.6)	70 (50)	52 (46.8)	20 (51.3)	14 (53.8)
Winter 2019, n (%)	165 (52.4)	70 (50)	59 (53.2)	19 (48.7)	12 (46.2)

Recruitment for the project was done via email communication, and both electronic and verbal consent were obtained. A total of 14 participants from the fall course offering and 12 participants from the winter course offering agreed to an interview. In addition, 6 key project stakeholders were recruited to participate in the evaluation. This subsample comprised 1 project leader, 1 project coordinator, 1 subject matter expert, 1 instructional designer, and 2 project advisory committee members. In addition, 5 nursing students who moderated the course discussion boards and offered support to participants through email were invited to provide qualitative feedback on web-based delivery via a focus group. Thus, the total sample size for the qualitative data was 37.

### Data Collection

A mixed methodology was used to evaluate web-based delivery of the course. Participants were asked to complete a web-based precourse survey that contained close-ended questions about their experience as a caregiver, their access to and use of technology, and demographic information for both themselves and their care recipient. Participants were then asked to complete a postcourse survey that contained the same questions as the precourse survey, with an extra section about their experience taking the course. Both quantitative surveys were administered anonymously on the web through LimeSurvey. Thus, participants’ confidentiality was maintained, as the answers could not be linked to individual participants.

Caregivers who finished most of the module content were invited to participate in one-on-one telephone interviews to provide more in-depth feedback. A semistructured interview guide with open-ended questions was used to ask participants about usability, accessibility, level of interaction, strengths, weaknesses, and areas of improvement regarding the *Caregiving Essentials* course. A total of 26 participant interviews were conducted. Qualitative feedback was also collected via telephone interviews with 6 key project stakeholders. This interview guide focused on the strengths, weaknesses, areas of improvement, and scalability of the course. In addition, a web-based focus group was conducted with 5 nursing students who played an active role in the course. Similarly, they were asked a combination of questions from both the participant and stakeholder interview guides.

### Data Analysis

The survey data collected from participants before and after the course could not be compared because there was a significant difference between the number of people who completed the precourse survey and those who completed the postcourse survey (Table 1). However, the postcourse survey responses were compared with the qualitative interview feedback and supported the major findings in terms of overlapping identified themes. Therefore, methodological triangulation was conducted by cross analyzing the 3 different forms of data collection. The survey data, interview data, and focus group data helped to ensure the validity of the key findings. The audio-recorded interviews and focus group were transcribed and analyzed using thematic coding in NVivo 12 Pro (QSR International). An inductive approach was used to identify 35 unique nodes and subfolders, which eventually led to the formation of overarching themes related to the main objective. These themes include accessibility to and within the course, level of interaction between peers and with the content, comfortability with and barriers to using technology, and scalability of the project.

Respondents were categorized based on their participant group (caregiver, stakeholder, or nursing student). If the participant was a caregiver, they were further categorized based on which course offering they took (Fall 2018 or Winter 2019). Therefore, the identifier F11 refers to a caregiver participant from the fall course offering, the identifier W2 refers to a caregiver participant from the winter course offering, S2 refers to a stakeholder participant, and NS5 refers to a nursing student participant.

## Results

### Participants

As noted in Table 2, slightly more than half (21/39, 54%) of those who participated in the postcourse survey (n=39) were aged between 45 years and 64 years, most self-identified as female (28/39, 72%), many (17/39, 44%) were providing care to a parent, about half (21/39, 53%) had been a caregiver for 1 to 3 years, almost half (19/39, 49%) were either employed part-time or full-time when they completed the survey, and one-third (13/39, 33%) reported providing informal care for more than 15 hours per week.

**Table 2:** Participant information from the postcourse survey (n=39).^a^

Postcourse survey questions and options		Participant, n (%)
**What is your age? (years)**		
	18-24	3 (8)
	25-34	1 (3)
	35-44	1 (3)
	45-54	7 (18)
	55-64	14 (36)
	65-74	3 (8)
	≥75	4 (10)
**What is your sex?**		
	Male	5 (13)
	Female	28 (72)
	Other	0 (0)
**What is your relationship with this person? Your care recipient is...**		
	Your parent	17 (44)
	Your spouse	7 (18)
	A family member	4 (10)
	A friend	0 (0)
	Other	5 (13)
**Approximately how many hours per week do you provide care to this person?**		
	1-4	6 (15)
	5-9	8 (21)
	10-14	5 (13)
	15-19	2 (5)
	≥20	11 (28)
**Are you currently employed?**		
	Yes: full-time	15 (38)
	Yes: part-time	4 (10)
	No	10 (26)
	Other, please specify	4 (10)
**How long have you been a caregiver? (years)**		
	<1	3 (8)
	1-3	21 (54)
	4-6	5 (13)
	≥7	0 (0)
**What is your estimated annual household income before taxes? (Can $ [US $])**		
	<15,000 (12,433.33)	0 (0)
	15,000-29,000 (12,433.33-24,037.76)	2 (5)
	30,000-49,999 (24,866.65-41,443.59)	9 (23)
	50,000-69,999 (41,444.42-58,021.36)	2 (5)
	70,000-99,999 (58,022.19-82,888.01)	5 (13)
	>100,000 (82,888.84)	6 (15)
	Prefer not to answer	9 (23)

^a^Response rate was not 100% for each question.

### Strengths of Web-Based Delivery

Most of the caregivers who participated in the evaluation component of the project stated that they preferred it over an in-person intervention. One participant said:

If I had to show up at a place, I probably would not have participated as much as being able to do it online.

W2

Similarly, another interviewee said:

The reason why I enrolled in this online course is because I’m extremely busy and I couldn’t always make it in person.

W8

One project stakeholder expressed their understanding of the importance of web-based delivery for the course:

People don’t want to come out or maybe they can’t get out because of that person that they have at home and it’s not easy to find some relief ... The online was just vital.

S2

These positive interview comments correlate with the high number of caregiver respondents who agreed (30/35, 86%) or somewhat agreed (4/35, 11%) to survey statement number 6 (“In the future, I would be willing to take an online course again.”), as shown in Table 3.

**Table 3:** Caregiver participant postcourse survey results (n=35).^a^

Item number	Survey statement	Agree, n (%)	Somewhat agree, n (%)	Disagree, n (%)
1	I would recommend this course to a friend.	34 (97)	1 (3)	0 (0)
2	I am comfortable sharing my ideas in written format online.	14 (40)	15 (43)	4 (11)
3	I am confident using and contributing to an online discussion group when I need help or information.	15 (43)	12 (34)	5 (14)
4	I feel comfortable assessing the information I discover online for their integrity and truthfulness.	17 (49)	15 (43)	0 (0)
5	I am satisfied with the level of interaction in this course.	26 (74)	6 (17)	2 (6)
6	In the future, I would be willing to take an online course again.	30 (86)	4 (11)	1 (3)

^a^Response rate was not 100% for all questions.

More specifically, several participants praised the flexibility of the course and their ability to participate wherever and whenever. One respondent noted:

The material ... lent itself well to doing things independent and online—which is what I was looking for.

F11

Quite a few caregivers spoke to the self-paced nature of the course, mentioning how the ability to “[do] it on my time” (W9) and “hop online anytime that works” (W4) was extremely valuable to them. One participant described how the flexibility of the course benefitted their level of access:

I could participate in the course at home, when I’m at school; it didn’t prevent getting access to the information in any way ... doing it online was the best option.

F3

Although some liked the fact that “[i]t’s in the comfort of your own house” (W7), others enjoyed the ability to log into the course from work “on and off throughout the day, and during my lunch breaks” (W3).

As one respondent put it:

It was a good way because ... for all the caregivers, we all have different times of when we’re available.

W10

This strength was realized and echoed by one stakeholder as well:

It was presented in a manner that would be palatable to older adults who are quite busy.

S5

Similarly, one member of the focus group of nursing students also agreed:

Having it on their own terms ... knowing they have it right in their own home, was valuable to them.

NS5

The flexibility of module information intake was highlighted as another important feature:

I liked how you could stop and play at your own pace.

W8

Another caregiver stated:

It was a good thing because you could go back if you forgot anything.

W12

Other participants talked about repetition in viewing module content:

I’ve gone through it a couple of times.

F5

I could go back and look at some of the modules I had already finished, just to kind of review.

W5

Others chose to only read through the information that was most relevant to them:

I kind of just scanned over ... really focused on the things that I needed.

F8

The control over choosing how much time to invest in the course and in each section of the modules seemed beneficial:

You can spend as much time or as little time on those modules as you like.

W2

An additional element of accessibility was the free course registration. A number of caregivers expressed appreciation for the affordability of the course in their interviews (F3, F8, W3, and W11). Accessibility was considered throughout the whole design process, as stated by one project stakeholder:

A distinct strength was that this was a “no cost,” open opportunity for caregivers. We worked hard to ensure there would be as few hurdles to access as many online materials as possible.

S4

Besides reducing financial barriers, the web-based aspect of *Caregiving Essentials* also helped to tackle geographical limitations:

Technology ... can facilitate crossing a barrier, including the barrier of geography ... Again, it ties into access.

S4

As one stakeholder stated:

It’s an online course and we very specifically reached out to people who were living in Northern Ontario.

S6

One interviewee spoke about the lack of accessibility of care resources in the North from personal experience:

...because of my northern roots and because I’m working up in education in the north, I knew that there’s a tremendous need for this kind of education.

S4

Web-based delivery ensured that even informal caregivers in remote regions of the province had equal access to the course. One participant specifically praised the project leadership for targeting recruitment efforts to Northern communities in Ontario:

I thought that was excellent because you’re reaching the people that are—there’s a whole bunch of need obviously ... They’re really isolated it feels.

F10

Another strength identified under accessibility was the user-friendliness of the course. One participant commented:

I was very impressed about how the course was set up, how easy it was to access, and how easy it was to maneuver through all the areas.

W1

Another remarked:

The navigation through the learning or training was straight forward, well labeled, the links all worked, everything was functional and very easy to use.

W12

The feedback from the nursing students involved in the course reflected participants’ comments:

The course is very easy to navigate ... it was really well organized.

NS4

One caregiver who initially experienced difficulties explained that the navigation became easier over time:

It took me a bit the first module to find out how to get to the next, but once I did that, it was okay.

W5

Therefore, it seems that both access to the web-based course itself as well as ease of accessing information within the course were 2 highlights of the user experience.

The discussion boards were another well-accepted element of web-based delivery, as reported by numerous participants. Many believed that the opportunity to connect with other participants was an important part of the course:

There was a common camaraderie. It was nice ... that you do have that option to connect.

W4

One caregiver described discussing shared experiences as *“*really comforting in a lot of ways” (F12).

For the less experienced caregivers who did not contribute to the discussion boards, some still found the posts to be “kind of refreshing to get the perspective that there’s lots of people out there dealing with this” (F11).

Newer caregivers were able to read posts from more experienced caregivers and consequently felt more prepared:

It’s more hearing what other people have to say and seeing what I have to look forward to ... or not look forward to.

W5

The benefits of the discussion boards were also realized by stakeholders and nursing students:

The idea that people could talk to each other, get to know each other, share stories with each other.

S6

Another interviewee made the following observation about the discussion board activity:

People were using it to either commiserate or to justify some of the decisions they are making as caregivers themselves.

S3

Likewise, they were described as “[r]eally important for the caregivers to feel that they were supported in their role, and kind of feeling that they weren’t alone” (NS3). Another focus group member said they were “essential to the course in order to relate with other caregivers” (NS2).

Aligning with the caregivers’ feedback, one nursing student described the sharing of experiences as creating “a sense of camaraderie” (NS4), whereas another referred to it as a “community with peer support” (NS5).

In addition, one student noted:

The discussion board gets interaction going ... different caregivers answer back ... help each other out.

NS1

Even among the caregivers who did not use the discussion boards, some still saw value in incorporating social interaction for others:

I never get involved with that kind of thing, but I think that’s great ... You don’t want to feel like, “Am I the only one going through this?”

W7

As mentioned in some of the caregiver interviews, part of the reason for lower participation in the discussion boards was simply personal preference or prioritizing learning from the modules over making new connections with others.

In terms of web-based delivery aspects that participants liked and would keep the same, 8 participants mentioned the postmodule quizzes, and 8 participants mentioned the web-based support relating to course information, information technology troubleshooting, and general questions. Regarding the self-check quizzes after each module, one nursing student expressed:

I really liked that it tested your knowledge.

NS2

Another student commented:

I think that the modules are already quite interactive when testing your knowledge.

NS4

During the focus group, the nursing students also described the value in caregivers having the option to reach out to them for help with the course:

I know the email was good too. They could directly contact us if they were having issues with IT, or if they had ... more sensitive issues that they wanted to discuss.

NS2

Thus, the more interactive elements of the course seemed to enhance the participants’ overall experience. This qualitative feedback corresponds with the postcourse survey results, as shown in Table 3. Most respondents agreed (26/35, 74%) or somewhat agreed (6/35, 17%) to survey statement number 5 (“I am satisfied with the level of interaction in this course.”).

Furthermore, the variety of resources used to deliver information was also identified as a positive factor:

I hadn’t encountered such a comprehensive collection of resources. Also, in terms of types of resources—so videos, documents, templates.

F12

Likewise, someone else highlighted this as a strength:

I liked the fact that there was a variety of different ways to get the information. You had the odd case study, you had a link to another website, ... downloadable files.

W12

One stakeholder also referenced this strong point:

The other thing that I think was really good about this project was that it brought a whole lot of different resources together in one place.

S6

### Barriers to Web-Based Delivery

Although many participants from all 3 groups cited accessibility as a major strength of web-based delivery, there were some who identified limitations with the navigation:

When I was going into a video or something, it would go into the video and then it was hard for me to go back.

W10

A different participant described a similar situation:

Certain links take you to other places and navigating to get back to the original place ... was a little bit challenging.

W4

Another caregiver also shared about some trouble with web-based functionality:

I had difficulty navigating out of the discussion board ... I would always end back at the home screen and then have to go back into the module.

F2

Someone else mentioned:

I’m pretty savvy with computers so it wasn’t so much that I didn’t know how to access it. I just found it a little bit clumsy with the windows and having to scroll down.

F4

One of the older caregiver participants remarked:

I didn’t try because I couldn’t figure out how to make it work.

F5

Older participants and/or those living in Northern areas face their own barriers to accessibility, as noted by one participant:

It’s unfortunate being online, there’s so many people in the community who don’t have internet or don’t have access to internet ... in Northern Ontario.

F13

One interviewee commented:

I have a computer, [but] a lot of people do not in my age bracket.

F4

Even when participants had access to a computer and the internet, a lack of comfort with using technology and web-based platforms proved to be another barrier to participation:

I am 75 ... Not everybody this age is limited in their computer experience, but unfortunately, I am one of them that is.

F5

Comparably, another person declared:

I’m 70, so I’m not as computer literate ... so things are a little more difficult for me.

W5

This limitation was also highlighted by one of the nursing students in the focus group:

Depending on how old the caregiver is, they may not be “technology acceptable,” or able in a way.

NS5

One of the students even said that they found that “the site isn’t the most intuitive” (NS4), which could make accessibility more of a challenge for certain participants, especially older ones.

Another barrier to participation in the web-based course was the lack of peer engagement experienced by some users. Certain individuals felt the discussion boards were lacking interaction between caregivers:

There weren’t many people at all engaged in sharing information, which is a shame because I think we’re all on the same journey.

W9

Someone else expressed the desire for lengthier conversations:

I would’ve liked to see a back and forth more with what people were saying ... I would’ve liked to have had more discussion on what other people’s opinions were.

W10

A caregiver described how a sense of community was not there for them:

One of the reasons I’d join the course was to perhaps be part of the community, be part of the tribe, dealing with the same issues. I just didn’t find that. People that perhaps did log in weren’t consistent in logging in. Or people that had very similar issues to what I was going through, I couldn’t find them again on various chat boards.

W11

Another caregiver cited the self-paced nature of the course as being problematic in this way as well:

I went through it faster than what was recommended ... so because of that, there was nothing in the online chat because other people hadn’t gotten there yet.

W5

One reason for the lack of discussion board participation was the concerns with sharing private information on the web:

I wasn’t ready to share on the internet.

F5

Another respondent reiterated this worry:

I wasn’t comfortable using my personal experience in an online public discussion.

W8

These comments were also reflected in the postcourse survey results, as shown in Table 3. Statement number 2 (“I am comfortable sharing my ideas in written format online.”) and number 3 (“I am confident using and contributing to an online discussion group when I need help or information.”) had the lowest participant agreement levels (14/35, 40% and 15/35, 43%, respectively).

### Suggestions to Improve Web-Based Delivery

Recommendations for improving engagement between participants included adding a discussion thread where caregivers could share resources (F3), creating small participation groups based on geographic location (W12), and using a telecommunication for live discussions (F3, F4, F7, F11, W3, and W9). Some people specifically referred to integrating videoconferencing and emphasized the significance of face-to-face interactions. However, as some participants had expressed security concerns, one caregiver’s idea could be used as a potential solution:

My name was on the post. Is there a way to make it anonymous or change your identity when commenting? My concern was anonymity for myself and for my family members.

W11

Not using full names or even using pseudonyms or usernames could also be applied to a video call feature as a way to maintain some aspect of privacy.

Some improvements for the discussion boards, as suggested by the project stakeholders, were using caregivers as moderators to offer more of a “peer-to-peer experience” (S1) and creating smaller discussion groups to “connect [those] who were living in the same areas” (S6).

Other ideas to enhance participant interaction were using additional communication methods, such as a web conference (S1) or audio-video chats (S4). One interviewee remarked that when “you can see someone’s face, and who they are, it makes a big difference” (S2).

Another recommended upgrade for web-based delivery was to organize the content so that more information is presented broadly via modules and so that each module contains more specific information through a series of different subsections (F1, F14, and W11). This structure would streamline content better and make it easier for caregivers to find what they are looking for. Some participants said that there was too much text to read (F3, F5, W4, and W10), and it was suggested to either add a feature that reads the text or include more video clips into the modules (F3). Other proposed enhancements were to offer a download option for the material (W11) and to include short testimonies from informal caregivers and/or older adults (W6).

The last theme that arose was the opportunity for future growth. A couple of caregiver participants recommended that the course should be opened to a broader and larger audience, such as other types of caregivers, caregivers living in other provinces, and other care workers (F10, W1, and W4). The web-based delivery of *Caregiving Essentials* would certainly enable scalability to the national level because geographical barriers are reduced. Course expansion was also brought up in several stakeholder interviews:

In terms of how the course is actually designed, it certainly could handle a larger audience.

S6

A total of 2 factors that would need to be addressed while scaling up the course would be ensuring that the information and resources in the modules are kept updated (S3 and S6) and remain region-specific (S2 and S6).

## Discussion

### Principal Findings

Many of the strengths and areas of improvement identified by the caregiver participants aligned with the feedback from the project stakeholders and nursing students. The web-based delivery of the *Caregiving Essentials* course enabled course accessibility for most of the informal caregivers who participated in the study. Stakeholders were aware of informal caregivers’ busy and often unpredictable schedules, so the course was designed to be flexible, which participants valued a great deal. The self-paced, independent nature of the course was made possible by web-based, stand-alone modules. Participants liked the fact that they could access the course from home, work, or school whenever they had free time. Some also found it helpful that they could pick and choose which information they wanted to focus on and could even go back to the review material if needed. This flexibility was highlighted as a benefit by the stakeholders and nursing students.

The reported strengths from the project evaluation align with the findings from the existing literature. In the evaluation of the Connect, Assess, Respond, Evaluate, and Share (CARES) Dementia Basics Program for caregivers by Pleasant et al [13], convenience, portability, and customizable learning speed are cited as advantages of web-based learning programs. Moreover, accessibility was identified as one of the main benefits of the web-based modality for an individual psychoeducational stress management training program offered on the web to family caregivers [14]. In addition, the convenience and suitability of asynchrony and the ability to personalize use were noted as favorable features of internet-based social support networks by caregivers of older adults [15].

Only 1 participant thought that the web-based delivery specifically hindered their course experience, which was due to their lack of experience with computers and technology. Others also shared some experiences of having difficulty navigating through certain areas of the course. Although several participants described the course as easily accessible, user-friendly, and straightforward, a few referred to sections of the course as being clumsy or sporadic. This variation in feedback may be caused by individual factors, such as familiarity with web-based courses or generational differences in the use of technology. The disparity in positive and negative responses can also be due to areas of the course that need to be improved to better suit the diverse needs of various users.

The discussion boards were another major strength identified by the stakeholders, nursing students, and participants, as they made the course more engaging. The course designers created discussion board topics that coincided with the module topics to encourage participant activity. The main goal of web-based communication was to increase interaction among users and to combat social isolation. Many participants reported a sense of community and camaraderie. The nursing students who moderated the discussion boards confirmed the positive connection that was building when they spoke about participants sharing stories and giving each other advice.

Connecting with other caregivers was also a strength observed in other studies. In a systematic review of web-based interventions for caregivers by Parra-Vidales et al [18], they found that allowing participants to have a direct web-based contact with other caregivers contributed to the effectiveness of the interventions. In the study by Barbabella et al [20] on a web-based psychosocial intervention for family caregivers of older people, findings revealed positive effects on social inclusion and support from the interactive services that enabled communication among participants. In the study by Godwin et al [21], all studies involving technology-driven interventions for caregivers that were reviewed had some positive findings, and each had an information and social support component.

Not all participants found the discussion boards to be beneficial. The postcourse survey results provided in Table 3 show that around half of the respondents were not confident in sharing their ideas in a written format on the web. This correlated with the participants who had privacy concerns and did not wish to share personal information on the web. Some participants found the discussion boards to be challenging to navigate, others prioritized exploring the module content, and a few accessed the discussion boards when there was little interaction. These experiences have been found elsewhere among caregivers of older adults. In the study by Colvin et al [15] on exploring computer-mediated communication, the complaints that arose included concerns around anonymity, a lack of adequate response, and a lack of privacy or confidentiality.

Other interactive features, such as the postmodule quizzes, the downloadable Caregiver Action Plan, and email support, were also said to enhance the overall experience of taking *Caregiving Essentials*. The positive feedback for these course components corresponds with the elements identified among other web-based interventions that have been shown to be effective in previous work. Boots et al [16] found that multicomponent internet interventions that combined tailored information with interactions among caregivers were the most promising for improvements. Similarly, in the systematic review of internet-based interventions for caregivers of older adults by Guay et al [17], a combination of interactive web-based activities and the provision of human support are 2 components that have been shown to contribute to intervention efficacy.

A couple of participants mentioned that they liked the various ways in which information was presented, although numerous people suggested that even more multimedia types should be added to the modules to help reduce the amount of onscreen text. Increasing the level of engagement was another recommendation made by the stakeholders, nursing students, and participants. Specific improvements that were suggested included adding web conference presentations, smaller group chats, and live video calling. Telecommunication applications such as Google Hangout and Skype were brought up, as many people emphasized the importance of face-to-face connections. This is consistent with the findings from the literature. In a qualitative study by Ploeg [28] on a web-based transition toolkit, My Tools 4 Care, participants suggested that adding a feature to enable caregivers to connect with one another (in real time or asynchronously) to share information, experiences, and caregiving strategies would be helpful. Furthermore, in comparing 2 internet-based intervention programs, Marziali and Garcia [10] found that the videoconferencing intervention program was deemed more useful in improving caregivers’ mental health status than the chat-based intervention. This is useful considering that the discussion boards within *Caregiver Essentials* were intended to reduce social isolation.

Another important theme was geography and the role it played throughout the project from the recruitment process to the data collection stage. Some of the participants were specifically recruited from Sudbury and Timmins in Northern Ontario, where there is a lack of resources and accessibility barriers for the ones that do exist. Therefore, the participants’ ability to access the course and their insights from the interviews about web-based delivery were especially appreciated because they represent an underserved subgroup among informal caregivers. Stakeholders belonging to the project leadership team were knowledgeable about service access limitations in Northern Ontario. Therefore, the web-based delivery of the course reduced spatial barriers and allowed for equal participation from caregivers, regardless of where they were located. The accessibility of the intervention to remote regions was also emphasized by Marziali and Donahue [22] in the pilot feasibility study on Caring for Others, an internet group intervention for family caregivers of older adults.

This is a key factor to recognize, especially if the project were to expand to other geographic areas. Using some of the domains of the nonadoption, abandonment, scale-up, spread, and sustainability framework, there are some characteristics of *Caregiving Essentials* that show promising results in terms of evaluating the potential for future effectiveness and success [29]. For the technology domain, the intervention lies somewhere between the simple and complicated categorization because some participants did not need a set of instructions to access and navigate the course, whereas others did make use of the detailed instruction and *helpdesk* support. For the value proposition domain, the technology is desirable for its intended users, safe, and cost-effective; therefore, it would lean more toward being labeled a simple innovation. For the last domain of the framework, there is a strong scope for adapting and embedding the technology as local need or context changes.

### Limitations

A limitation of the evaluation was the recruitment strategies used to recruit participants. Only caregivers who had finished most of the module content were contacted for an interview. Therefore, if participants stopped partway through, they were never given the opportunity to provide in-depth feedback pertaining to the web-based delivery of the course. The topic of evaluation is one in which participants would likely still be able to provide feedback on if they had completed at least one module and had explored other features of the course. Thus, it is possible that participants who qualified to be involved in the evaluation (ie, finishing most of the module content) were more likely to offer certain types of responses. This means that the participant interview data may not accurately represent the perspectives of everyone who took the course.

Furthermore, the voluntary aspect of the project’s evaluation is another potential factor that may reduce the generalizability of the participant interview findings. Again, individuals who agreed to provide feedback may be more likely to hold extreme opinions, whether positive or negative. Moreover, as the evaluation was not mandatory, the number of participants who completed each step decreased throughout the duration of the project. If participation in the *Caregiving Essentials* course was tied to participants’ commitment to provide evaluative feedback, then there may not have been such a loss in numbers between the pre- and postcourse surveys.

### Conclusions

In conclusion, this evaluation of web-based delivery of the *Caregiving Essential* course demonstrated acceptability and usability for many of the participants. A diverse range of accessibility topics and the ways in which they enabled participation in the course were discussed in the stakeholder and participant interviews and the student focus group. Suggestions to further develop the existing interactive features of the intervention were made, as well as recommendations to incorporate additional methods of engagement via technological opportunities were provided. Although there were some barriers to participation due to web-based delivery, most respondents were able to overcome them and still benefit from the course. Web-based delivery of the knowledge intervention had many advantages and positively impacted informal caregivers’ experiences in taking the course. The proposed areas of improvement offered feasible changes, and several changes were implemented for future course offerings following the evaluation.

Further use or investigation is warranted to evaluate the effectiveness of web-based delivery for this course and other existing and emerging web-based interventions for informal caregivers of older adults. This population experiences a great need for credible, relevant, and up-to-date information and resources. It is key that the web-based modalities of interventions for caregivers enhance accessibility and enable meaningful human interactions. The findings from this evaluation can support the creation and improvement of the current and new interventions. It can also be applied to innovations related to other populations that provide care to older adults.

## Abbreviations

CARES: Connect, Assess, Respond, Evaluate, and Share
