# Correction: Web-Based Delivery of the Caregiving Essentials Course for Informal Caregivers of Older Adults in Ontario: Mixed Methods Evaluation Study

**DOI:** 10.2196/42215

**Published:** 2022-08-31

**Authors:** Shelley Rottenberg, Allison Williams

**Affiliations:** 1 School of Earth, Environment & Society McMaster University Hamilton, ON Canada

In “Web-Based Delivery of the Caregiving Essentials Course for Informal Caregivers of Older Adults in Ontario: Mixed Methods Evaluation Study” (JMIR Aging 2021;4(2):e25671), the authors made a change in the corresponding author's contact information.

In the corrected version, the corresponding author’s phone number has been updated to “1 905 525 9140.”

The correction will appear in the online version of the paper on the JMIR Publications website on August 31, 2022, together with the publication of this correction notice. Because this was made after submission to PubMed, PubMed Central, and other full-text repositories, the corrected article has also been resubmitted to those repositories.

